# P-Glycoprotein–Mediated Efflux Reduces the In Vivo Efficacy of a Therapeutic Targeting the Gastrointestinal Parasite *Cryptosporidium*

**DOI:** 10.1093/infdis/jiz269

**Published:** 2019-06-08

**Authors:** Samuel L M Arnold, Ryan Choi, Matthew A Hulverson, Grant R Whitman, Molly C Mccloskey, Carlie S Dorr, Rama S R Vidadala, Mansi Khatod, Mary Morada, Lynn K Barrett, Dustin J Maly, Nigel Yarlett, Wesley C Van Voorhis

**Affiliations:** 1 Division of Allergy and Infectious Disease, Department of Medicine, University of Washington, Seattle; 2 Center for Emerging and Reemerging Infectious Disease, University of Washington, Seattle; 3 Department of Chemistry, University of Washington, Seattle; 4 Department of Biochemistry, University of Washington, Seattle; 5 Pace University, New York, New York

**Keywords:** *Cryptosporidium*, gastrointestinal, drug development, P-gp, drug efflux, enteric

## Abstract

Recent studies have illustrated the burden *Cryptosporidium* infection places on the lives of malnourished children and immunocompromised individuals. Treatment options remain limited, and efforts to develop a new therapeutic are currently underway. However, there are unresolved questions about the ideal pharmacokinetic characteristics of new anti-*Cryptosporidium* therapeutics. Specifically, should drug developers optimize therapeutics and formulations to increase drug exposure in the gastrointestinal lumen, enterocytes, or systemic circulation? Furthermore, how should researchers interpret data suggesting their therapeutic is a drug efflux transporter substrate? In vivo drug transporter–mediated alterations in efficacy are well recognized in multiple disease areas, but the impact of intestinal transporters on therapeutic efficacy against enteric diseases has not been established. Using multiple in vitro models and a mouse model of *Cryptosporidium* infection, we characterized the effect of P-glycoprotein efflux on bumped kinase inhibitor pharmacokinetics and efficacy. Our results demonstrated P-glycoprotein decreases bumped kinase inhibitor enterocyte exposure, resulting in reduced in vivo efficacy against *Cryptosporidium*. Furthermore, a hollow fiber model of *Cryptosporidium* infection replicated the in vivo impact of P-glycoprotein on anti-*Cryptosporidium* efficacy. In conclusion, when optimizing drug candidates targeting the gastrointestinal epithelium or gastrointestinal epithelial infections, drug developers should consider the adverse impact of active efflux transporters on efficacy.

## Teaser

How intestinal P-glycoprotein drug efflux affects therapeutics targeting intestinal pathogens was not clear. For bumped kinase inhibitors targeting *Cryptosporidium*, P-glycoprotein reduces the therapeutic enterocyte exposure, and this is associated with reduced in vivo efficacy in a mouse model of cryptosporidiosis.

*Cryptosporidium* is a protozoan parasite that can cause debilitating, moderate-to-severe diarrhea. Infection with *Cryptosporidium* is associated with stunting and death in children <2 years old [1–7]. Therapeutic options for cryptosporidiosis are limited, and the only Food and Drug Administration–approved treatment, nitazoxanide, has only marginal efficacy against cryptosporidiosis in both malnourished children and immunocompromised adults [8–10]. Given the clear need for new treatment options, academic laboratories and pharmaceutical companies have been working together to develop novel anti-*Cryptosporidium* treatments. As part of this collaborative effort, a target product profile was developed to outline the desired characteristics for an anti-*Cryptosporidium* therapeutic [11]. Among the target product profile criteria, the guidelines describing the desired systemic drug exposure have been an ongoing topic of discussion [12, 13]. While this discourse continues in the anti-*Cryptosporidium* drug-development community, in vivo evidence with at least 1 series of anti-*Cryptosporidium* compounds, bumped kinase inhibitors (BKIs), suggests gastrointestinal (GI) drug exposure is the main contributor to in vivo efficacy, and therapeutics localized to the systemic circulation may only have a minor role in the observed effect [14].

**Figure 1.**
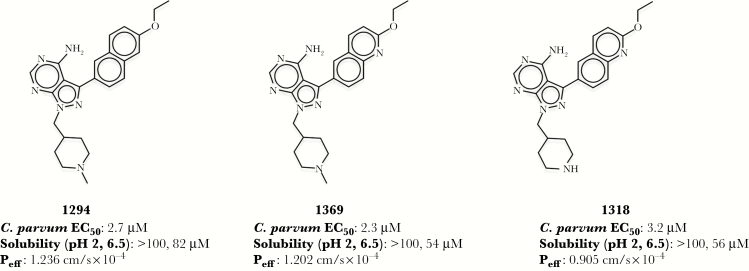
Structures, in vitro efficacy, and physicochemical properties of bumped kinase inhibitors (BKIs). Structures of all 3 BKIs are based on a 1 H-pyrazolo{2,3-d}pyrimidin-4-amine scaffold. BKI1369 and BKI1318 only differ by a methyl group on the N-pyridine of BKI1369, and BKI1294 has a naphthalene ring, compared with the quinoline ring of BKI1369 and BKI1318. The *Cryptosporidium parvum* half-maximal effective concentration (EC_50_) is nearly identical across BKIs. Solubilities for each BKI were measured at pH 2 and 6.5, to represent the stomach and small intestinal environments, respectively. The human effective permeability (P_eff_) was predicted as described in Materials and Methods.

**Figure 2.**
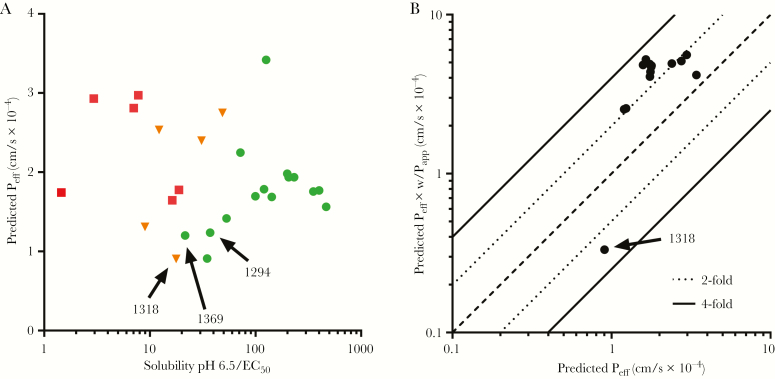
The solubility and permeability of bumped kinase inhibitors (BKIs). *A*, For BKIs previously evaluated in a mouse model of *Cryptosporidium* infection, the predicted permeability values were plotted against the BKI in vitro solubility (pH 6.5)/in vitro BKI half-maximal effective concentration against *C. parvum*. Each point represents a BKI, and the shape (color) refers to the in vivo efficacy of the compound: circle (green), >2-log reduction in *C. parvum*; triangle (orange), 0–2-log reduction in *C. parvum*; and square (red), no reduction in *C. parvum*. *B*, In addition, to determine how the incorporation of Caco-2–derived apparent permeability (P_app_) data affected the effective permeability (P_eff_) predictions, the P_eff_ predicted with Caco-2 in vitro P_app_ data was plotted against the predicted permeability without in vitro data. The data represent a subset of BKIs that were evaluated in the mouse model of cryptosporidiosis. Except for BKI1318, the inclusion of the Caco-2–derived P_app_ resulted in predicted P_eff_ values 2–4-fold greater than those generated without Caco-2 data.

BKIs are among the leading candidates under development for the treatment of cryptosporidiosis, with established efficacy in mouse, calf, and piglet disease models [15, 16]. One of the first BKI candidates developed for the treatment of cryptosporidiosis, BKI1294, demonstrated remarkable efficacy in neonatal and adult mouse models of *Cryptosporidium parvum* infection [15]. Despite relatively low plasma exposure with oral dosing, BKI1294 continued to demonstrate efficacy in a calf model of cryptosporidiosis [16]. Unfortunately, owing to cardiotoxicity, BKI1294 development was discontinued. Based on the GI localization of *Cryptosporidium*, the development of potential BKI drug candidates includes a large focus on compound solubility and permeability because these properties control drug absorption into the GI epithelium. Therefore, successors to BKI1294 were designed to have similar permeability and solubility characteristics. Analogs of BKI1294 with nearly equivalent structures, BKI1318 and BKI1369, were progressed through mouse efficacy experiments in a previously published study [15]. The structural similarity shared between the 3 BKIs results in comparable in vitro solubility, predicted permeability, and in vitro efficacy against *C. parvum* (Figure 1). However, when BKI1318 and BKI1369 were evaluated in a mouse model of cryptosporidiosis, only BKI1369 generated efficacy analogous to that of BKI1294, with a >2-log *C. parvum* reduction (Figure 2A) [15]. While the factor(s) contributing to the variability in efficacy was unclear, it was hypothesized that the predicted effective permeability (P_eff_) was not capturing the difference in GI absorption of the BKIs in vivo.

While in silico models to predict P_eff_ will incorporate the predicted impact of passive transport, the models will not include the potential impact of active transport. Active influx and efflux transporters may significantly alter the absorption of therapeutics in the GI tract, leading to unpredictable outcomes [17]. While it is well understood that drug efflux may diminish the efficacy of therapeutics targeting cancer cells or the central nervous system by decreasing drug exposure at the site of action, how drug transporters may affect the treatment of enteric diseases is unclear. As the infectious diseases community actively works on the development of a new cryptosporidiosis treatment, there are lingering questions on the desired localization of drug within the GI tract (lumen versus enterocyte). Previous work has provided evidence suggesting that enterocyte exposure is associated with BKI efficacy [14]. These results align with the observed localization of *Cryptosporidium* species to intracellular but extracytosolic parasitophorous vacuoles located beneath the apical plasma membrane of infected intestinal epithelial cells [18, 19]. In this scenario, enterocyte drug exposure is generating the desired in vivo efficacy, and any active drug efflux by a drug efflux transporter localized to the intestinal apical brush-border epithelial cells may diminish BKI efficacy (eg, P-glycoprotein [P-gp]). To determine whether an active efflux transporter was responsible for the lack of BKI1318 in vivo efficacy, studies were performed with both in vitro and in vivo models. Caco-2 studies were used to characterize the in vitro apparent permeability (P_app_) of BKIs and to identify BKI1318 as a substrate of P-gp efflux. Pharmacological inhibition of P-gp was used to confirm active BKI1318 efflux was associated with altered plasma and GI levels of BKI. Finally, the impact of P-gp–associated efflux on BKI efficacy was confirmed with both in vitro and in vivo models of *Cryptosporidium* infection.

## MATERIALS AND METHODS

Information regarding *C. parvum* growth inhibition by BKIs, the hollow fiber model, liquid chromatography–tandem mass spectrometry (LC-MS/MS) methods, and the interferon γ (IFN-γ) knockout (KO) mouse model of *C. parvum* infection has been described in detail previously [15, 20–23]. Information on BKI permeability, LC-MS/MS quantification of elacridar, and quantification of *Cryptosporidium* by polymerase chain reaction analysis is described in the Supplementary Materials. Unless noted otherwise, statistical significance was determined with an unpaired Student *t* test, using a significance level of 0.05.

### BKI Efficacy With Hollow Fiber In Vitro *Cryptosporidium* Culture

*C. parvum* was cultured using a hollow fiber bioreactor modified for evaluation of potential chemotherapeutic compounds [21–23]. Briefly, the system used a 20-kD MWCO polysulfone fiber cartridge (FiberCell Systems, Frederick, MD). HCT-8 cells were grown on the outer surface of the fibers (extracapillary space), and reservoirs were used to provide nutrients to the cells. When the glucose concentration decreased to at least 2 g/L, the extracapillary space was inoculated with 10^6^*C. parvum* (Iowa isolate, Bunch Grass Farms, Deary, ID). When the *C. parvum* yield reached 10^8^ parasites/mL, BKI1369, BKI1294, BKI1318, elacridar, or a solution containing both BKI1318 and elacridar was added via the extracapillary port to a nominal concentration of 40 μM (BKI1294) or 80 μM (BKI1369, BKI1318, and elacridar). Compound concentration was reduced over time by the flow of medium through the extracapillary and intracapillary spaces. Samples were removed from the extracapillary space once per day for evaluation of parasite numbers.

### In Vivo Efficacy in a Mouse Model of *Cryptosporidium* Infection

Efficacy in female IFN-γ KO mice (B6.129S7-Ifngtm1Ts/J; Jackson Laboratories) aged 8–10 weeks was evaluated as previously described [15]. Beginning on day 6 after infection, mice were dosed with 30 mg/kg BKI1318 suspended in 0.2 mL of vehicle, 15 mg/kg elacridar in 0.1 mL of vehicle followed 15 minutes later by 30 mg/kg BKI1318 in 0.1 mL of vehicle, or 0.2 mL of vehicle only once daily for 5 days. Fecal specimens were collected daily from each group and weighed. Each fecal sample was checked for luminescence on the day of collection, and the number of relative light units was determined and normalized to fecal sample weights.

### Animal Ethics Statement

All animal experiments were approved by the Institutional Animal Care and Use Committee at the University of Washington. All animals used in this study were handled in strict accordance with practices made to minimize suffering and the number of animals used.

## RESULTS

### Permeability of BKIs

While the predicted P_eff_ values were nearly identical for all 3 BKIs, the measured in vitro P_app_ value for BKI1318 was much lower than for BKI1369 and BKI1294. Caco-2 Transwell assays provide a well-documented model for accessing compound permeability and were used to characterize the in vitro P_app_ of BKI1294, BKI1369, and BKI1318 (Supplementary Table 1). The apical to basolateral permeability (A → B) represents absorption of drug from the GI lumen (apical permeability) into the portal vein (basolateral permeability). The A → B P_app_ was similar for BKI1369 and BKI1294, but the permeability observed for BKI1318 was approximately 50-fold lower. When the A → B and B → A were compared to calculate the efflux ratio, the value for BKI1318 was approximately 30-fold greater than those for BKI1369 and BKI1294 (Figure 2A). Next, to determine how the Caco-2 data affected the prediction of intestinal P_eff_, the P_eff_ of multiple BKIs was predicted both with and without the incorporation of Caco-2–derived P_app_ values (Figure 2B). The inclusion of P_app_ data generated P_eff_ values within 4-fold of those predicted without Caco-2 data. Except for BKI1318, the P_eff_ was increased by the incorporation of Caco-2 data. Although these data suggest that an efflux transporter may be reducing the GI absorption of BKI1318, they do not definitively identify the transporter contributing to the efflux.

Given the well-known role of P-gp in the efflux of many diverse drugs, the adenosine triphosphate–binding cassette (ABC) transporter was specifically chosen for initial studies, to identify the transporter responsible for BKI1318’s relatively low P_app_. A Caco-2 system with overexpression of P-gp was used to verify the role of P-gp in BKI1318 transport (Supplementary Table 2). While the efflux ratios were approximately 1 for BKI1369 and BKI1294, the efflux ratio was 19 for BKI1318 and 45 for the positive control, digoxin, a known substrate of P-gp. In addition, the known P-gp inhibitor, valspodar, lowered BKI1318’s efflux ratio from 19 to 1. Taken together, these data suggest that P-gp is the transporter responsible for the active transport of BKI1318. It should be noted that this experiment characterized the importance of P-gp with a single concentration of BKI1318. The kinetics of BKI1318 transport mediated by P-gp are still unknown, and it is not clear how the fraction of absorption mediated by P-gp transport will change with different concentrations of BKI1318.

### Alterations in Plasma BKI Pharmacokinetics by P-gp Inhibition

**Figure 3.**
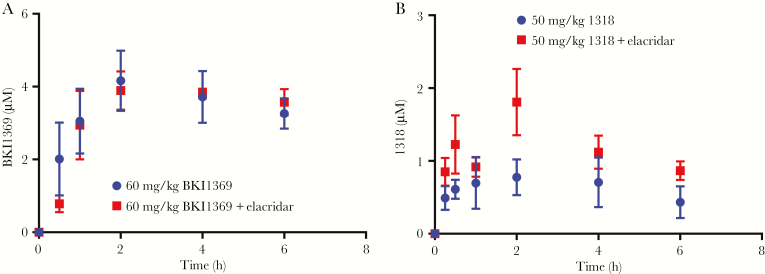
In vivo P-glycoprotein (P-gp) inhibition increases bumped kinase inhibitor 1318 (BKI1318) plasma concentrations. *A*, When the P-gp inhibitor elacridar was coadministered with BKI1369, plasma concentrations were not significantly different than those observed with BKI1369 dosed alone. *B*, However, the observed plasma maximum concentration and area under the curve of the P-gp substrate BKI1318 were significantly increased by coadministration of elacridar (*P* < .05). Each point represents the average plasma concentration (±standard deviation) observed in 3 mice.

Based on the impact of P-gp on the in vitro permeability of BKI1318 as compared to observations for BKI1369 and BKI1294, the coadministration of a selective in vivo P-gp inhibitor, elacridar, was investigated to confirm P-gp–mediated efflux of BKI1318 in vivo. Coadministration of elacridar was predicted to increase the systemic levels of BKI1318 but not BKI1369 after oral administration to mice. A 30 mg/kg-dose of elacridar was administered orally 15 minutes before oral administration of 60 mg/kg BKI1369 or 50 mg/kg BKI1318 (Figure 3). When elacridar was administered before BKI1369, there was not a significant change in the pharmacokinetics (Supplementary Table 3). In contrast, elacridar coadministration significantly increased both the maximum concentration (C_max_) and the area under the curve from 0 to ∞ (AUC_0–∞_) of BKI1318 by approximately 2-fold (*P* < .05). While elacridar increased the BKI1318 plasma AUC_0–∞_, the BKI1318 oral clearance (calculated as [clearance/bioavailability]) observed with elacridar coadministration (207 mL/hour) was still 5-fold greater than observed for BKI1369, suggesting efflux alone was not the only contributor to the difference in observed exposure for the BKIs.

### BKI In Vitro Efficacy Against *Cryptosporidium* With Polarized and Nonpolarized HCT-8 Cells

To evaluate the potential impact of P-gp efflux on BKI1318 in vitro efficacy, in vitro assays with polarized and nonpolarized HCT-8 cells were evaluated. The nonpolarized HCT-8 coculture assay to screen anti-*Cryptosporidium* drug candidates has been previously reported by us in detail [15]. When 200 nM or 2000 nM elacridar was coadministered with a BKI1318 dose response, there was no difference in the observed BKI1318 half maximal effective concentration (EC_50_). Based on the reported elacridar half maximal inhibitor concentration for the inhibition of P-gp, the P-gp activity was predicted to be reduced with both elacridar concentrations. The EC_50_ values (±standard errors) for BKI1318, BKI1318 plus 200 nM elacridar, and BKI1318 plus 2000 nM elacridar were 1.6 ± 1.5, 2.1 ± 1.7, and 1.3 ± 1.3, respectively. Elacridar alone had no effect on *C. parvum* levels.

**Figure 4.**
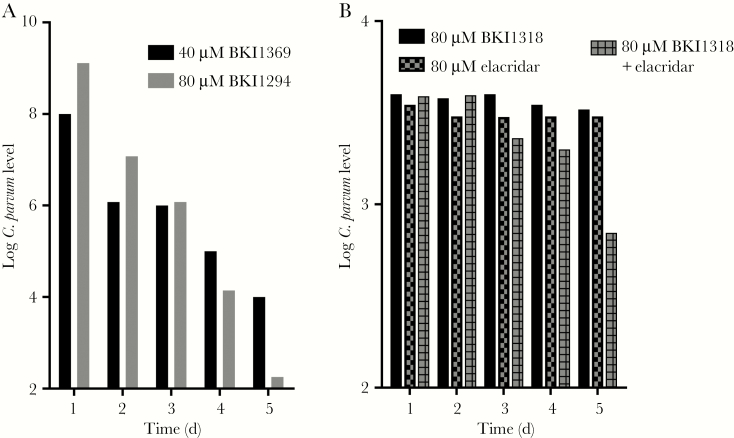
Effect of bump kinase inhibitors (BKIs) on *Cryptosporidium parvum* growth in a hollow fiber culture system. *A*, When BKI1294 was administered at 80 µM once per day for 4 days, there was a >6-log reduction in the *C. parvum* level by 24 hours after the final dose, on day 4. Similar efficacy was observed when 40 µM BKI1369 was administered once daily for 4 days. *B*, Unlike BKI1294 and BKI1369, 80 µM BKI1318 administered once daily had no effect on *C. parvum* growth in the hollow fiber model. However, coadministration of the P-glycoprotein inhibitor elacridar (80 µM) with BKI1318 generated an approximately 1-log reduction in *C. parvum* levels. Elacridar alone had no effect on *C. parvum* in the hollow fiber model. Each point represents the number of *C. parvum* oocysts in a single 0.5-mL sample collected at each time point from the extracapillary space.

To evaluate the efficacy of BKIs against *C. parvum* with polarized HCT-8 cells, a hollow fiber coculture model was used to characterize the efficacy of BKI1294, BKI1369, and BKI1318. The hollow fiber model consists of dual compartments generating an environment representative of the GI tract. HCT-8 cells polarize on the fibers within the cartridge of the hollow fiber system, and *C. parvum* interacts with the apical side of the HCT-8 cells. When the BKI efficacy was evaluated in this model over a time course, BKI1369 and BKI1294 reduced *C. parvum* levels by >3 orders of magnitude over the course of the study (Figure 4A). However, in a separate study, BKI1318 did not decrease the levels of *C. parvum* (Figure 4B). To determine whether P-gp inhibition would increase the efficacy of BKI1318 observed with the hollow fiber model, elacridar was coadministered with BKI1318. While *C. parvum* levels were not reduced to the same extent as those observed with BKI1294 and BKI1369, the coadministration of elacridar with BKI1318 reduced *C. parvum* levels by an order of magnitude. In agreement with the results observed in the assay with nonpolarized HCT-8 cells, elacridar alone had no effect on *C. parvum* levels.

### GI Pharmacokinetics of BKI1318

**Figure 5.**
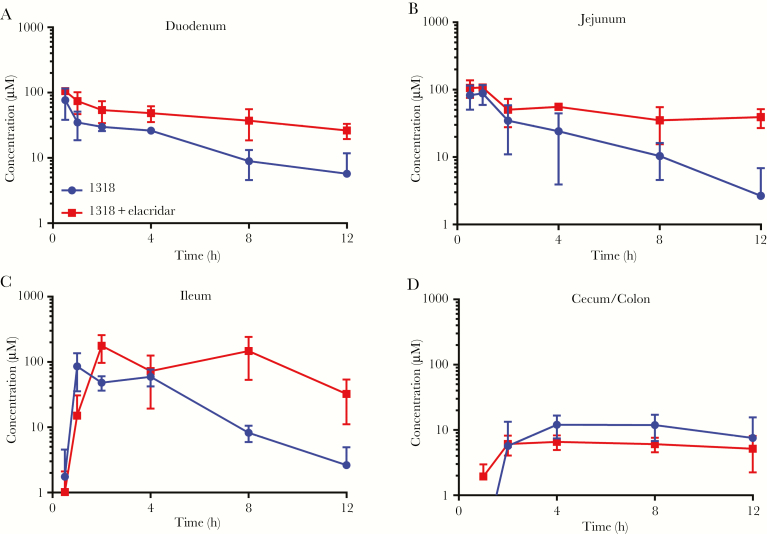
In vivo P-glycoprotein inhibition increases bumped kinase inhibitor 1318 (BKI1318) gastrointestinal concentrations. Elacridar (15 mg/kg) or vehicle control were administered to mice 15 minutes before delivery of a single oral dose of BKI1318 (30 mg/kg). Gastrointestinal tissue specimens were collected 0.5, 1, 2, 8, and 12 hours after BKI1318 dosing, and the lumen of the tissue was flushed extensively. *A*–*C*, Tissue concentrations of BKI1318 were quantified, and elacridar coadministration significantly increased the BKI1318 area under the curve in the duodenum (*P* < .001; *A*), jejunum (*P* < .05; *B*), and ileum (*P* < .05; *C*). *D*, However, elacridar did not generate a significant difference in the BKI1318 exposure in the cecum/colon. Each point represents the average gastrointestinal concentration (±standard deviation) observed in 3 mice. An unpaired Student *t* test was used to determine significance, using a significance level of 0.05.

To determine whether P-gp inhibition increases BKI1318 enterocyte concentrations, BKI1318 GI concentrations were measured after mice received vehicle or 30 mg/kg elacridar 15 minutes before a single oral 50 mg/kg BKI1318 dose (Figure 5). At multiple time points following BKI1318 administration, mice were euthanized, and the GI tract was separated into segments corresponding to the duodenum, jejunum, ileum, and cecum/colon. After the luminal contents were thoroughly flushed, the tissue concentrations were determined for both BKI1318 and elacridar (Supplementary Tables 4 and 5). Similar to the results observed in plasma, BKI BKI1318 exposure was significantly higher (by approximately 2-fold; *P* < .05) in the duodenum and jejunum when elacridar was administered before the BKI. Although BKI1318 exposure increased by approximately 3-fold in the ileum (*P* < .05), elacridar did not significantly affect BKI1318 levels in the cecum/colon.

### BKI1318 Demonstrates In Vivo Anti-*Cryptosporidium* Efficacy When P-gp Is Inhibited

**Figure 6.**
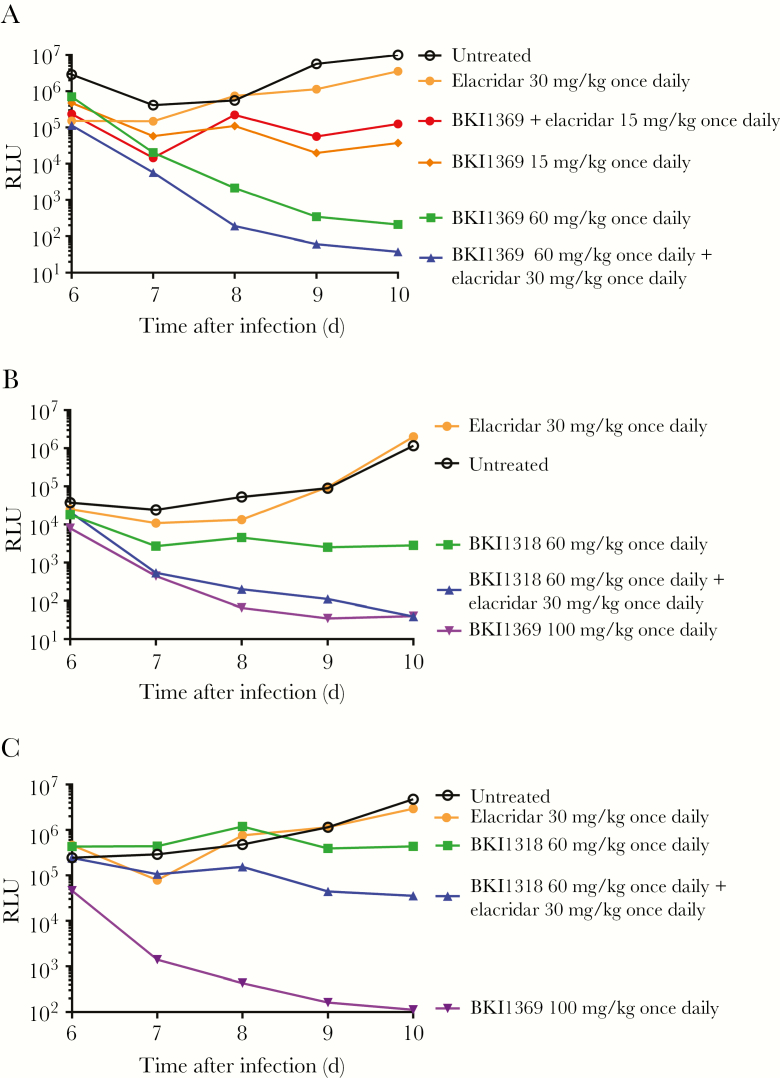
P-glycoprotein (P-gp) inhibition increases the in vivo efficacy of bumped kinase inhibitor 1318 (BKI1318). *A*, The in vivo P-gp inhibitor elacridar did not affect the in vivo efficacy of 15 mg/kg or 30 mg/kg BKI1369 administered once daily for 5 days in the mouse model of *Cryptosporidium* infection. *B*, In agreement with previous studies, the <1-log reduction in *C. parvum* oocyst excretion with 60 mg/kg BKI1318 was less than observed with 60 mg/kg BKI1369 administered once daily for 5 days. However, the coadministration of elacridar with BKI1318 generated an improvement in efficacy, demonstrating that P-gp was contributing to the reduced efficacy of BKI1318 in vivo. *C*, The elacridar influence on BKI1318 efficacy was confirmed by repeating the in vivo efficacy experiment. In agreement with findings of the initial experiment (shown in panel *B*), elacridar coadministration improved the observed BKI1318 efficacy. While the coadministration of elacridar with BKI1318 did not generate efficacy similar to that of 100 mg/kg BKI1369, which may have been due to the relatively low starting infection in the 100 mg/kg BKI1369 group. As shown in all panels, elacridar administration alone had no effect on *Cryptosporidium* infection. Each point represents the relative luminescence units (RLUs) observed for the pooled feces from each group (n = 3 mice/group).

Previous work with BKI1369 and BKI1318 established that the BKIs have different efficacy in an IFN-γ KO mouse model of cryptosporidiosis [15]. To determine whether the active efflux of BKI1318 was associated with the difference in efficacy between the compounds, the mouse model was used to evaluate the effect of elacridar coadministration on the in vivo efficacy (Figure 6). In agreement with the previous experiments, the efficacy of 60 mg/kg BKI1318 was less than that of 60 mg/kg BKI1369 when the BKIs were dosed without elacridar (Figure 6A and 6B). Furthermore, elacridar coadministration had no effect on the in vivo efficacy of BKI1369 (Figure 6A). However, coadministration of elacridar increased the level by which BKI1318 reduced the *Cryptosporidium* burden (Figure 6B). To confirm the elacridar-mediated increase in BKI1318 efficacy, the in vivo assay was repeated, with similar results (Figure 6C).

## Discussion

While the P-gp–mediated efflux of therapeutics targeting the central nervous system and cancer cells has been previously studied in detail, this is the first study investigating the effect of intestinal P-gp efflux on a therapeutic targeting an enteric pathogen. The BKIs evaluated in this study are leading candidates under development for the treatment of the GI-localized parasite *Cryptosporidium*. Previously, minor changes in BKI structure have been associated with large variations in their anti-*Cryptosporidium* in vivo efficacy, despite analogous solubility and comparable in vitro efficacy against *Cryptosporidium*. Based on the belief that absorption into the GI epithelium is essential for the in vivo efficacy of BKIs, BKI permeation was investigated as the main contributor to the variability in the in vivo efficacy. The predicted permeabilities were nearly identical for the evaluated BKIs, but in silico predictions of permeability do not account for the potential impact of drug transporters. While it seemed unlikely that such a modest change in BKI structure could dictate affinity for an active transporter, previous studies have observed enantiomer-selective differences in the transporter-mediated efflux of macrolides [24] and verapamil [25]. Based on the limitations of in silico predictions of permeability, Caco-2 cells were used to confirm that the permeability of BKI1318 in vitro was significantly lower than that of BKI1294 and BKI1369, suggesting that absorption of BKI1318 into the GI epithelium would be relatively lower. Further work with Caco-2 cells confirmed BKI1318 as a P-gp substrate, and multiple in vitro *Cryptosporidium* models were used to investigate whether the P-gp effect on BKI efficacy could be evaluated in vitro.

In contrast to a 2-dimensional (2D) in vitro model with nonpolarized HCT-8 cells, a hollow fiber model of *Cryptosporidium* infection with polarized HCT-8 cells produced in vitro efficacy results that aligned with the observed BKI in vivo efficacy. 2D HCT-8 models are commonly used to screen anti-*Cryptosporidium* drug candidates, and monolayers of HCT-8 cells have been confirmed to lack P-gp expression [26]. Unlike 2D HCT-8 models, the hollow fiber HCT-8 model has a redox environment representative of the GI lumen. HCT-8 cells polarize in the hollow fiber model, and polarized HCT-8 cells have been previously shown to express active P-gp [27]. Therefore, the identical efficacy observed for BKI1294, BKI1369, and BKI1318 in 2D HCT-8 models may be due to the lack of P-gp activity. These results suggest that 2D HCT-8 culture models provide useful information on the efficacy of therapeutics against *Cryptosporidium*, but they may not capture the effect of drug transporters, which may adversely impact the in vivo efficacy. Therefore, developers of anti-*Cryptosporidium* therapeutics are encouraged to screen lead candidates with an in vitro cell culture efficacy model expressing relevant efflux transporters or to screen for active transport in relevant in vitro permeability models. For the BKIs evaluated in this study, the Caco-2 assay and hollow fiber model demonstrated P-gp was affecting the in vitro permeability and efficacy of BKI1318, but it was not clear whether P-gp efflux significantly influenced the disposition and efficacy of BKI1318 in vivo.

In agreement with the Caco-2 in vitro data, coadministration of a P-gp inhibitor significantly increased systemic and GI levels of BKI1318. It has been previously shown that interactions with P-gp may lead to changes in regional drug distribution, attenuating efficacy without significant changes in systemic exposure [28]. Given the localization of *Cryptosporidium* to enterocytes, increased systemic exposure may not significantly improve in vivo efficacy. Previous work with BKIs demonstrated that, when given as congruent doses, oral dosing generates a larger reduction in *Cryptosporidium* levels in mice, compared with subcutaneous dosing [15]. Therefore, while the systemic BKI1318 C_max_ and AUC were significantly higher in mice coadministered elacridar, the minor increases were not expected to meaningfully improve the in vivo efficacy. Unlike systemic BKI concentrations, GI BKI exposure has been shown to be associated with in vivo efficacy, and coadministered elacridar significantly increased BKI1318 exposure in the small intestine, which is the predominant location of *Cryptosporidium* colonization in mice [29]. BKI1318 cecum/colon levels were not significantly higher with elacridar coadministration, and the difference between the small and large intestine exposure may be due to diminished P-gp inhibition, because elacridar levels were significantly lower in the cecum/colon, compared with the small intestine.

**Figure 7.**
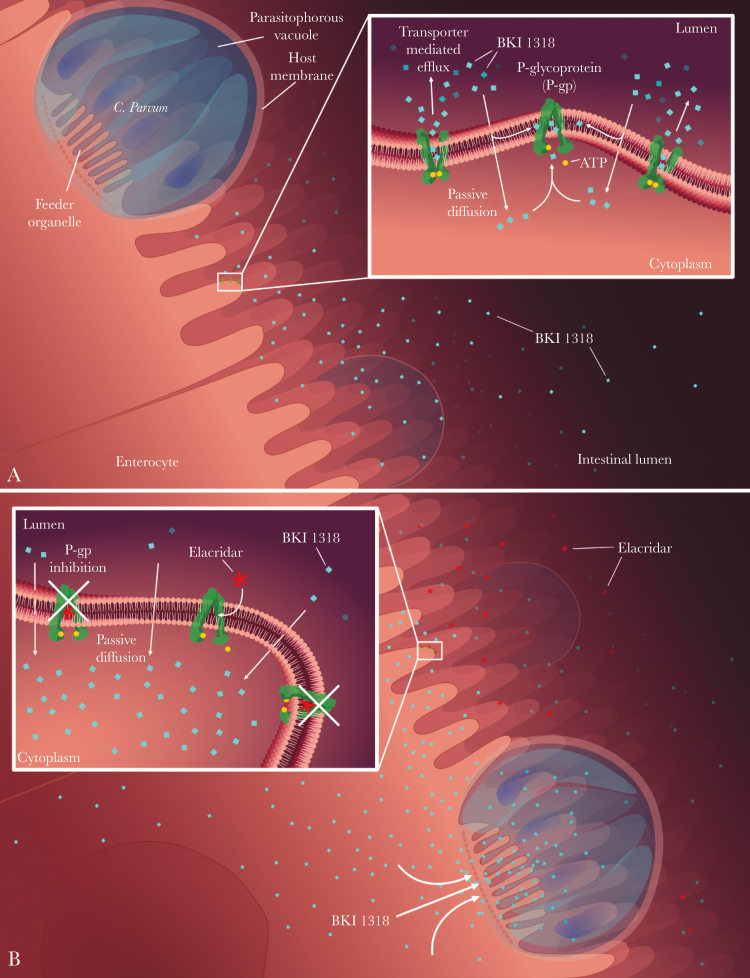
Illustration of the P-glycoprotein (P-gp) interaction with bumped kinase inhibitors (BKIs) in the gastrointestinal tract. *A*, Low absorption of BKI1318 into the enterocyte as a result of P-gp–mediated efflux. *B*, In the presence of the P-gp inhibitor elacridar, BKI1318 absorption into the intestinal epithelium allows for targeting of *Cryptosporidium*.

In association with the increased BKI1318 GI exposure, P-gp inhibition improved BKI1318 efficacy in a mouse model of *Cryptosporidium* infection. The improved outcome with elacridar coadministration is likely due to the increased BKI1318 enterocyte exposure, because intestinal P-gp efflux will reduce the exposure of *Cryptosporidium* to BKI1318 (Figure 7). As expected, since BKI1369 was not a P-gp substrate, the efficacy of BKI1369 was not affected by elacridar coadministration. When previously evaluated in a neonatal mouse model of *Cryptosporidium* infection, BKI1294, BKI1369, and BKI1318 were all efficacious [15]. While these efficacy results are not in agreement with those from the adult mouse model, the discrepancy may be due to age-specific differences in P-gp expression. It has been previously shown that mouse intestinal P-gp expression increases significantly with maturation [30].

In addition to adversely affecting therapeutics targeting cancer, P-gp is known to have a role in the disposition of human immunodeficiency virus protease inhibitors, antibiotics, and antifungals [31–34]. While inhibition of P-gp–mediated drug efflux in the GI tract, blood-brain barrier, and kidney is targeted to increase tissue drug concentrations, drug efflux transporters expressed by bacteria, fungi, and protozoa can also modulate drug disposition, leading to decreased efficacy [35–37]. *Cryptosporidium* organisms reside in a vacuole in the cytoplasm at the tip of enterocytes (Figure 7). A feeder organelle separates *Cryptosporidium* from the host cell, and this folded membrane structure regulates access to both nutrients and drugs. It is possible that an additional ABC transporter expressed along the feeder organelle is controlling BKI exposure to *Cryptosporidium* [38, 39]. The *Cryptosporidium Cp*ABC1 and *Cp*ABC2 transporters have many features resembling the MRP subfamily of ABC proteins [40, 41]. Elacridar has relatively minor activity against human MRP transporters as compared to P-gp, so it is assumed that elacridar most likely does not significantly inhibit *Cp*ABC1-2 [42]. Unlike *Cp*ABC1 and *Cp*ABC2, *Cp*ABC3 groups with the MDR transporter subfamily, suggesting it may be inhibited by elacridar [41]. However, no differences in BKI efficacy are observed in the nonpolarized HCT-8 infection model, suggesting there are no differences in BKI efflux by *Cryptosporidium* transporters. While the nonpolarized HCT-8 cells lack P-gp expression, *Cryptosporidium* transporter activity and substrate specificity should be retained in the model.

The results of this work further support the importance of drug absorption into the intestinal epithelium for therapeutics targeting *Cryptosporidium*. Before this study, it was not clear whether P-gp efflux would enhance or diminish the activity of a therapeutic targeting *Cryptosporidium*. If luminal drug exposure is responsible for the majority of in vivo efficacy against *Cryptosporidium*, P-gp efflux would potentially improve efficacy by increasing drug exposure along the GI lumen. However, the diminished relative efficacy of BKI1318 mediated by P-gp efflux illustrates the importance of drug absorption into the epithelium. Owing to differences in drug transporter kinetics, the results observed for BKI1318 may not translate to other anti-*Cryptosporidium* drug candidates that test positive as in vitro P-gp substrates. Novartis’ drug candidate KDU731, under development for the treatment of cryptosporidiosis, was reported to be a possible P-gp substrate, with a Caco-2 efflux ratio approximately half of that observed for BKI1318 [29]. However, KDU731 demonstrated excellent efficacy in multiple animal models, and this may be due to the different contributions of P-gp to KDU731 and BKI1318 absorption in vivo. Based on the clear impact of P-gp on BKI efficacy against *Cryptosporidium* infection in vivo, groups developing anti-*Cryptosporidium* therapeutics should carefully consider the factors contributing to the permeation of their drug candidates. While these studies specifically focused on the treatment of *Cryptosporidium* infection, the results of this work are applicable to drug-development programs targeting other enteric diseases.

## Supplementary Data

Supplementary materials are available at *The Journal of Infectious Diseases* online. Consisting of data provided by the authors to benefit the reader, the posted materials are not copyedited and are the sole responsibility of the authors, so questions or comments should be addressed to the corresponding author.

jiz269_Suppl_Supplementary_Material

jiz269_Suppl_Supplementary_Table_1

jiz269_Suppl_Supplementary_Table_2

jiz269_Suppl_Supplementary_Table_3

jiz269_Suppl_Supplementary_Table_4

jiz269_Suppl_Supplementary_Table_5

## Notes

***Financial support.*** This work was supported by the Bill and Melinda Gates Foundation (award OPP1160955 to S. A.), a grant to PATH from the Government of the United Kingdom (award DFI.1850-02-01291794-SUB to S. A.), the National Institute of Allergy and Infectious Diseases of the National Institutes of Health (awards R01AI089441, R01AI111341, and R01HD080670 to W. V. V.), and the US Department of Agriculture National Institute of Food and Agriculture (award 2014-06183 to W. V. V.).

***Potential conflicts of interest.*** W. C. V. V. is the president of ParaTheraTech, a company involved in developing BKIs for use in animal health. All other authors report no potential conflicts. All authors have submitted the ICMJE Form for Disclosure of Potential Conflicts of Interest. Conflicts that the editors consider relevant to the content of the manuscript have been disclosed.
