# Supplementary material for: P-Glycoprotein–Mediated Efflux Reduces the In Vivo Efficacy of a Therapeutic Targeting the Gastrointestinal Parasite Cryptosporidium
Source: J Infect Dis. 2019 Jun 8;220(7):1188–98. doi: 10.1093/infdis/jiz269 (PMC6736360; doi:10.1093/infdis/jiz269)
Supplement: jiz269_Suppl_Supplementary_Material [file jiz269_suppl_supplementary_material.docx]

Major Article: P-glycoprotein mediated efflux reduces the in vivo efficacy of a therapeutic targeting the gastrointestinal parasite *Cryptosporidium*

Samuel L. M. Arnold^1*^, Ryan Choi^1^, Matthew A. Hulverson^1^, Grant R. Whitman^1^, Molly C. McCloskey^1^, Carlie S. Dorr^1^, Rama S. R. Vidadala^2^, Mansi Khatod^3^, Mary Morada^3^, Lynn K. Barrett^1^, Dustin J. Maly^2,4^, Nigel Yarlett^3^, Wesley C. Van Voorhis^1^

^1^Department of Medicine, Division of Allergy and Infectious Disease, Center for Emerging and Reemerging Infectious Disease (CERID), University of Washington, Seattle, WA 98109 USA

^2^Department of Chemistry, University of Washington, Seattle, WA 98195 USA

^3^Pace University, New York, NY 10038 USA

^4^Department of Biochemistry, University of Washington, Seattle, WA 98195 USA

*Co-corresponding Authors: Samuel Arnold, phone, 206-616-7982 fax 206-616-4898 e-mail, slarnold@uw.edu

**Supplemental Methods and Materials**

**Permeability of BKIs**

To initially characterize the permeability of BKIs 1294, 1369, and 1318, the software MedChem Designer (Simulations +, Lancaster, CA) was used to predict the P_eff_. The in vitro apparent permeability (P_app_) was carried out by Absorption Systems LP (Exton, PA) with a Caco-2 trans-well assay. Caco-2 cells (clone C2BBe1) were obtained from American Type Culture Collection (Manassas, VA) were grown to confluence on collagen-coated, microporous membranes in 12-well assay plates. The permeability assay buffer was Hanks’ balanced salt solution (HBSS) containing 10 mM HEPES and 15 mM glucose at a pH of 7.4. The buffer in the receiver chamber also contained 1% bovine serum albumin. The dosing solution concentration was 5 µM BKI in assay buffer, and the cell monolayers were dosed on the apical side (A🡪B) or basolateral side (B🡪A) and incubated at 37°C with 5% CO_2_ in a humidified incubator. After 120 minutes, samples were taken from the donor and receiver chambers in duplicate. The P_app_ was calculated with equation (1) and the percent recovery with equation (2).

P_app_ = (*d*C_r_/*d*t) × V_r_/(A × C_A_) (1)

Percent Recovery = 100 × ((V_r_ × C_r_^final^) + (V_d_ × C_d_^final^))/(V_d_ × C_N_) (2)

where dC_r_/*d*t is the slope of the cumulative concentration in the receiver compartment versus time in µM s^-1^; V_r_ is the volume of the receiver compartment in cm^3^; V_d_ is the volume of the donor compartment in cm^3^; A is the area of the insert (1.13 cm^2^ for 12-well); C_A_ is the average of the nominal dosing concentration and the measured 120 minute donor concentration in µM; C_N_ is the nominal concentration of the dosing solution in µM; C_r_^final^ is the cumulative receiver concentration in µM at the end of the incubation period; C_d_^final^ is the concentration of the donor in µM at the end of the incubation period. All recovery values were >70% except for BKI 1294 A🡪B (62%).

For Caco-2 assays, the observed atenolol and propranolol P_app_ were ≤ 0.05 and 10-30 x 10^-6^ cm/s, respectively. To ensure no damage was inflicted to the cell monolayers during the flux period, lucifer yellow flux was also measured post-experimentally for each monolayer. To determine whether BKIs 1294, 1369, and 1318 were substrates of P-gp in vitro, P-gp efflux was characterized using the Caco-2 permeability assay protocol with a few modifications. The dosing solution concentration was 5 μM of BKI in the assay buffer +/- 1 μM valspodar. Cells were first pre-incubated for 30 minutes with HBSS containing +/- 1 μM valspodar. Cell monolayers were dosed on the apical side (A🡪B) or basolateral side (B🡪A), and incubated at 37°C with 5% CO_2_ in a humidified incubator. Samples were taken from the donor and receiver chambers at 120 minutes. Each determination was performed in duplicate.

**Compound detection and quantification by LC-MS/MS**

All samples from the pharmacokinetic experiments were analyzed with an Acquity UPLC in tandem with a Waters Xevo TQ-S micro (Waters, Milford, MA). The solvents were A: water with 0.1% formic acid and B: acetonitrile with 0.1% formic acid. Sample concentrations were determined using internal standard normalized calibration curves. Tissue and plasma from vehicle control treated mice were used as blanks or standards. Calibration curves for BKI 1318 and elacridar were prepared separately by adding 2 μL BKI to generate nominal concentrations of 0.004, 0.008, 0.02, 0.04, 0.08, 0.2, 0.4, 0.8, 2, 5, 10, and 20 μM. Calibration curves were generated separately for plasma and each section of the GI tract. On the day of extraction, 5 μL of plasma or tissue homogenate was added to a 96-well plate that contained 95 μL of 80:20 acetonitrile: water. As an internal standard, BKI 1517 was added to each sample to a final concentration of 20 nM. The samples were mixed and centrifuged at 4,700 rpm for 10 minutes. 100 μL of supernatant from each sample was placed in a 96-well plate, sealed, and analyzed by LC-MS/MS. All analyses were run with electrospray ionization in positive ionization mode and solvent gradient at curve of 6: 0 minutes 95%A, 5%B; 1 minute 95%A, 5%B; 5 minutes 5%A, 95%B; 6.5 minutes 5%A, 95%B; 6.6 minutes 95%A 5%B; 8 minutes 95%A 5%B. BKIs 1369, 1318, and elacridar analytes were monitored and quantified with the *m/z* transitions 418.2🡪112.0, 404.2🡪97.6, and 564.4🡪252.1, respectively. BKI 1517 was used as an internal standard and monitored with the *m/z* transition 354.2🡪281.1.

**In vivo BKI pharmacokinetics**

Mouse oral pharmacokinetic (PK) studies were performed as previously described^1^. Briefly, blood samples were taken by tail bleeding into heparinized tubes at designated time points and centrifuged to obtain plasma. The samples were frozen and stored at -20°C until extraction and analysis. PK calculations of T_max_, C_max_, AUC, oral clearance, and half-life were performed using Pharsight Phoenix WinNonlin software (Certara, St. Louis, MO).

Concentrations of BKI 1318, BKI 1369, and elacridar in the duodenum, jejunum, ileum, and cecum/ascending colon were measured as previously described^2^. Briefly, mice were administered a single 30 mg/kg oral dose of BKI 1318 alone, or a single dose of 15 mg/kg elacridar followed 15 minutes later by a single 30 mg/kg oral dose of BKI 1318. At 0.5, 1, 2, 4, 8, and 12 hours, three mice in each group were sacrificed by cervical dislocation, and the duodenum, jejunum, ileum, and cecum/ascending colon were immediately collected. Each section of the GI tract was flushed with Dulbecco's phosphate-buffered saline, weighed, and placed in storage at -80°C. On the day of homogenization, 0.9% NaCl was added to each sample to achieve 200 mg/mL tissue concentration. Samples were homogenized using a handheld motorized homogenizer (Fisher Scientific, Waltham, MA) on ice and stored at -20°C until extraction and analysis. To report tissue concentrations, 1 gram of tissue was estimated to be 1 mL of volume.

**Determination of parasite numbers by PCR in the hollow fiber culture system**

*C. parvum* growth was determined by analysis of the amount of *C. parvum* 18S-rRNA present and compared to the HCT-8 18S-rRNA as previously described^3^. Briefly this was performed by centrifugation at 14,000 x g for 5 min of 0.5 mL samples collected from the extracapillary space (ECS). The pellet was resuspended in 100 μL of iScript buffer and subject to 6-freeze/thaw cycles comprising 1 min in liquid nitrogen followed by 1.5 min at 70^o^C. The lysate was collected by centrifugation at 16,162 x *g* for 5 min, and the RNA purified according to the manufacturer’s instructions (iScriptTM, RT-qPCR sample preparation kit, Bio-Rad Laboratories). *C. parvum* was enumerated by quantitating the *Cp*18S-rRNA using the following specific forward and reverse primers - *Cp*18S-995F: 5’-TAGAGATTGGAGGTTCCT-3’ and *Cp*18S-1206R: 5’-CTCCACCAAC TAAGAACGCC-3’ and compared to the human *Hs*18S-rRNA using the following primer sets - *Hs*18S-F1373: 5’-CCGATAACGAACGAGACACTCTGG-3’ and *Hs*18S-R1561: 5’-TAGGGTAG GCACACGCTGAGCC-3’. The amount of 18S-rRNA was determined using 5 ng samples by RT-qPCR as described the manufacturer instructions using a Luna Universal One-Step^®^ RT-qPCR kit (New England Biologics) and a Quant Studio 6 Flex 44 instrument. The cell number is calculated from the C_T_ means using a set of standards prepared from 10^4^, 10^5^, and 10^6^ *C. parvum* oocysts and compared to 10^4^, 10^5^, and 10^6^ HCT-8 cells.

**References**

1 Tatipaka, H. B. *et al.* Substituted 2-phenylimidazopyridines: a new class of drug leads for human African trypanosomiasis. *J Med Chem* **57**, 828-835, doi:10.1021/jm401178t (2014).

2 Arnold, S. L. M. *et al.* Necessity of Bumped Kinase Inhibitor Gastrointestinal Exposure in Treating Cryptosporidium Infection. *J Infect Dis* **216**, 55-63, doi:10.1093/infdis/jix247 (2017).

3 Zhang, H., Guo, F. & Zhu, G. Cryptosporidium Lactate Dehydrogenase Is Associated with the Parasitophorous Vacuole Membrane and Is a Potential Target for Developing Therapeutics. *PLoS Pathog* **11**, e1005250, doi:10.1371/journal.ppat.1005250 (2015).
