# Supplementary material for: P-Glycoprotein–Mediated Efflux Reduces the In Vivo Efficacy of a Therapeutic Targeting the Gastrointestinal Parasite Cryptosporidium
Source: J Infect Dis. 2019 Jun 8;220(7):1188–98. doi: 10.1093/infdis/jiz269 (PMC6736360; doi:10.1093/infdis/jiz269)
Supplement: jiz269_Suppl_Supplementary_Table_1 [file jiz269_suppl_supplementary_table_1.pdf]

Supplemental Table 1: Bumped kinase inhibitor Caco-2 apparent permeability (P_{app}) and efflux ratio

Compound	Direction	P_{app} (10^{-6} cm/s)	Efflux Ratio
**BKI 1369**	A→B	12.4	2.6
	B→A	32.7	
**BKI 1318**	A→B	0.29	69
	B→A	20.3	
**BKI 1294**	A→B	12.7	1.8
	B→A	22.6	
**Propranolol**	A→B	23.2	Not applicable
**Atenolol**	A→B	0.21	Not applicable
