# Supplementary material for: P-Glycoprotein–Mediated Efflux Reduces the In Vivo Efficacy of a Therapeutic Targeting the Gastrointestinal Parasite Cryptosporidium
Source: J Infect Dis. 2019 Jun 8;220(7):1188–98. doi: 10.1093/infdis/jiz269 (PMC6736360; doi:10.1093/infdis/jiz269)
Supplement: jiz269_Suppl_Supplementary_Table_2 [file jiz269_suppl_supplementary_table_2.pdf]

Supplemental Table 2: Caco-2 assay to determine bumped kinase inhibitor P-gp substrate classification

Compound	Direction	Efflux Ratio	P-gp Substrate Classification
**BKI 1369**	A→B / B→A	1.6	Negative
**BKI 1369 + 1 µM Valspodar**	A→B / B→A	0.8	
**BKI 1318**	A→B / B→A	19	Positive
**BKI 1318 + 1 µM Valspodar**	A→B / B→A	1.1	
**1294**	A→B / B→A	1.5	Negative
**BKI 1294 + 1 µM Valspodar**	A→B / B→A	0.9	
**Digoxin**	A→B / B→A	45	Positive
**Digoxin + 1 µM Valspodar**	A→B / B→A	1.6	
