# Supplementary material for: P-Glycoprotein–Mediated Efflux Reduces the In Vivo Efficacy of a Therapeutic Targeting the Gastrointestinal Parasite Cryptosporidium
Source: J Infect Dis. 2019 Jun 8;220(7):1188–98. doi: 10.1093/infdis/jiz269 (PMC6736360; doi:10.1093/infdis/jiz269)
Supplement: jiz269_Suppl_Supplementary_Table_3 [file jiz269_suppl_supplementary_table_3.pdf]

Supplemental Table 3: Pharmacokinetics of bumped kinase inhibitors with co-administration of elacridar.

BKI	C_{max} (µM)	C_{max} w/ Elacridar (µM)	T_{max} (hours)	T_{max} w/ Elacridar (hours)	$AUC_{0\text{-}inf}$ (µmol*hr/L)	$AUC_{0\text{-}inf}$ w/ Elacridar (µmol*hr/L)
1369 60 mg/kg	4.2± 0.7	4.1 ± 0.3	2.0 ± 0.0	4.0 ± 1.6	75.6 ± 8.9	91.4 ± 5.0
1318 50 mg/kg	1.0 ± 0.1	1.8 ± 0.4*	2.3 ± 1.2	2.0 ± 0.0	6.6 ± 1.7	12.0 ± 1.6*

Note- BKI, Bumped kinase inhibitor; AUC, Area under the curve. *P<0.05
