# Supplementary material for: P-Glycoprotein–Mediated Efflux Reduces the In Vivo Efficacy of a Therapeutic Targeting the Gastrointestinal Parasite Cryptosporidium
Source: J Infect Dis. 2019 Jun 8;220(7):1188–98. doi: 10.1093/infdis/jiz269 (PMC6736360; doi:10.1093/infdis/jiz269)
Supplement: jiz269_Suppl_Supplementary_Table_4 [file jiz269_suppl_supplementary_table_4.pdf]

Supplemental Table 4: Bumped kinase inhibitor 1318 pharmacokinetics in the gastrointestinal tract with co-administration of elacridar

GI Segment	Observed C_{max} (µM)	C_{max} w/ Elacridar (µM)	T_{max} (hours)	T_{max} w/ Elacridar (hours)	AUC_{0-12} (µmol*hr/L)	AUC_{0-12} w/ Elacridar (µmol*hr/L)
Duodenum	77.2 ± 31.7	109.5 ± 3.7	0.5 ± 0.0	0.7 ± 0.2	235.4 ± 27.1	536.7 ± 45.1***
Jejunum	108.3 ± 5.4	98.1 ± 33.1	0.8 ± 0.2	1.8 ± 1.5	279.3 ± 65.1	558.4 ± 106.1*
Ileum	79.8 ± 29.1	177.3 ± 65.8	4.3 ± 2.9	2 ± 0.0	336.3 ± 76.1	1149.5 ± 496.4*
Cecum/Colon	14.4 ± 4.1	8.2 ± 0.3	9.3 ± 1.9	7.3 ± 4.1	108.8 ± 35.6	65.5 ± 4.0

Note- BKI, Bumped kinase inhibitor; AUC, Area under the curve. *P≤0.05,**P≤0.01,***P≤0.001
