# Supplementary material for: P-Glycoprotein–Mediated Efflux Reduces the In Vivo Efficacy of a Therapeutic Targeting the Gastrointestinal Parasite Cryptosporidium
Source: J Infect Dis. 2019 Jun 8;220(7):1188–98. doi: 10.1093/infdis/jiz269 (PMC6736360; doi:10.1093/infdis/jiz269)
Supplement: jiz269_Suppl_Supplementary_Table_5 [file jiz269_suppl_supplementary_table_5.pdf]

Supplemental Table 5: Elacridar pharmacokinetics in the gastrointestinal tract

GI Segment	Observed C_{max} (µM)	T_{max} (hours)	AUC_{0-12} (µmole*hr/L)
Duodenum	61.2 ± 20.9	0.6 ± 0.2	118.0 ± 4.9
Jejunum	32.6	0.6 ± 0.2	103.9 ± 21.4
Ileum	35.2 ± 10.3	1.7 ± 0.5	170.0 ± 45.7
Cecum/Colon	3.3 ± 3.1	6.0 ± 4.3	12.6 ± 8.5
